# Unresectable gastric cancer with gastric outlet obstruction and distant metastasis responding to intraperitoneal and folfox chemotherapy after palliative laparoscopic gastrojejunostomy: report of a case

**DOI:** 10.1186/1477-7819-8-109

**Published:** 2010-12-20

**Authors:** Joong-Min Park, Kyong-Choun Chi

**Affiliations:** 1 Department of Surgery, Chung-Ang University College of Medicine, Seoul, Korea; 2 Chung-Ang University Yongsan Hospital, 65-207 Hangangro-3-ga, Yongsan-gu, Seoul, 140-757, Korea

## Abstract

**Background:**

Gastric outlet obstruction (GOO) caused by unresectable gastric cancer is a challenging aspect of patient care. There have been no reports involving patients with obstructing gastric cancer and several incurable factors curatively treated by multimodal treatments.

**Case presentation:**

We report a case of 55-year-old man who was diagnosed with a poorly differentiated adenocarcinoma in the pre-pyloric antrum with GOO by gastroscopy. An abdominal computed tomography (CT) scan revealed thickening of the gastric wall and adjacent fat infiltration, and a large amount of food in the stomach suggesting a passage disturbance, enlarged lymph nodes along the common hepatic and left gastric arteries, and multiple hepatic metastases. The serum carcinoembryonic antigen (CEA) level was 343 ng/ml and the carbohydrate antigen (CA) 19-9 level was within normal limits. The patient underwent a laparoscopic gastrojejunostomy for palliation of the GOO. On the 3^rd ^and 12^th ^days after surgery, he received intraperitoneal chemotherapy with 40 mg of docetaxel and 150 mg of carboplatin. Simultaneously, combined chemotherapy with 85 mg/m^2 ^of oxaliplatin for the 1^st ^day and 600 mg/m^2 ^of 5-FU for 2 days (FOLFOX regimen) was administered from the 8^th ^post-operative day. After completion of nine courses of FOLFOX, the patient achieved a complete response (CR) with complete disappearance of the primary tumor and the metastatic foci. He underwent a radical subtotal gastrectomy with D3 lymph node dissection 4 months after the initial palliative surgery. The pathologic results revealed no residual primary tumor and no lymph node metastasis in 43 dissected lymph nodes. He has maintained a CR for 18 months since the last operation.

**Conclusion:**

Combination chemotherapy with systemic and intraperitoneal chemotherapy following laparoscopic bypass surgery showed marked efficacy in the treatment for unresectable advanced gastric cancer with GOO.

## Background

Although survival of patients with gastric cancer after surgery has been improved by early detection and curative surgery, the prognosis of patients with highly advanced gastric cancer, especially with distant metastasis such as peritoneal dissemination or hematogenous metastasis, is usually very poor. Chemotherapy is the treatment of choice for metastatic advanced gastric cancer; however, a standard treatment regimen has not been established. Neoadjuvant chemotherapy for advanced gastric cancer with or without distant metastasis has been reported [1-4]. For patients with peritoneal dissemination, intraperitoneal chemotherapy is an additional treatment option [1,5]; however, only a few long-term survivors have been reported after intraperitoneal chemotherapy.

Gastric outlet obstruction (GOO) caused by unresectable antral gastric cancer is another challenging aspect of patient care. Treatment of GOO is important for the patient with unresectable gastric cancer who needs chemotherapy. There have been no reports involving patients with gastric cancer and GOO and several incurable factors curatively treated by multimodal treatments.

In this report, we describe our experience of a patient with unresectable gastric cancer with GOO and multiple metastases who achieved a histologic complete response (CR) to intraperitoneal and FOLFOX chemotherapy after palliative laparoscopic gastrojejunostomy.

## Case report

**Figure 1:**
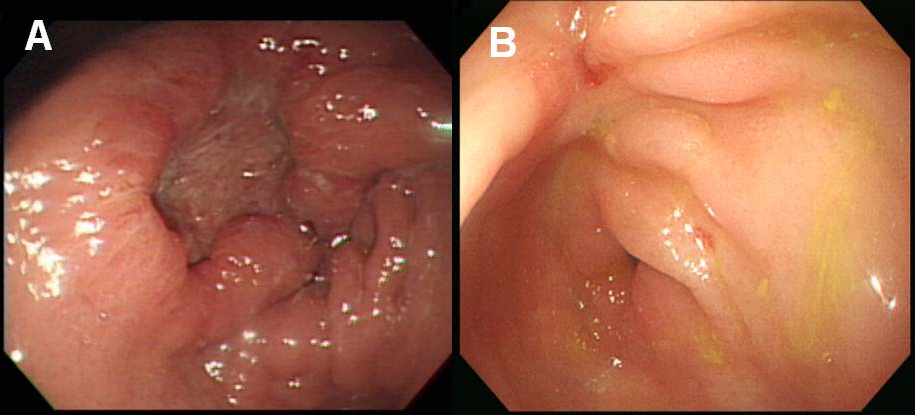
**Gastroscopy (A) before and (B) after chemotherapy**. (A) Gastroscopy demonstrated a deep ulcerative lesion in the lesser curvature side of the pre-pyloric antrum. The biopsy specimen showed poorly differentiated adenocarcinoma. (B) The primary tumor had changed to an ulcerated scar. The finding of gastric outlet obstruction disappeared.

A 55-year old man visited our hospital for evaluation of epigastric pain and poor oral intake of 2 months duration. A gastroscopy demonstrated a deep ulcerative lesion in the lesser curvature side of the pre-pyloric antrum and a large amount of food retained in the stomach because of GOO (Figure 1A). The biopsy revealed poorly differentiated adenocarcinoma.

**Figure 2:**
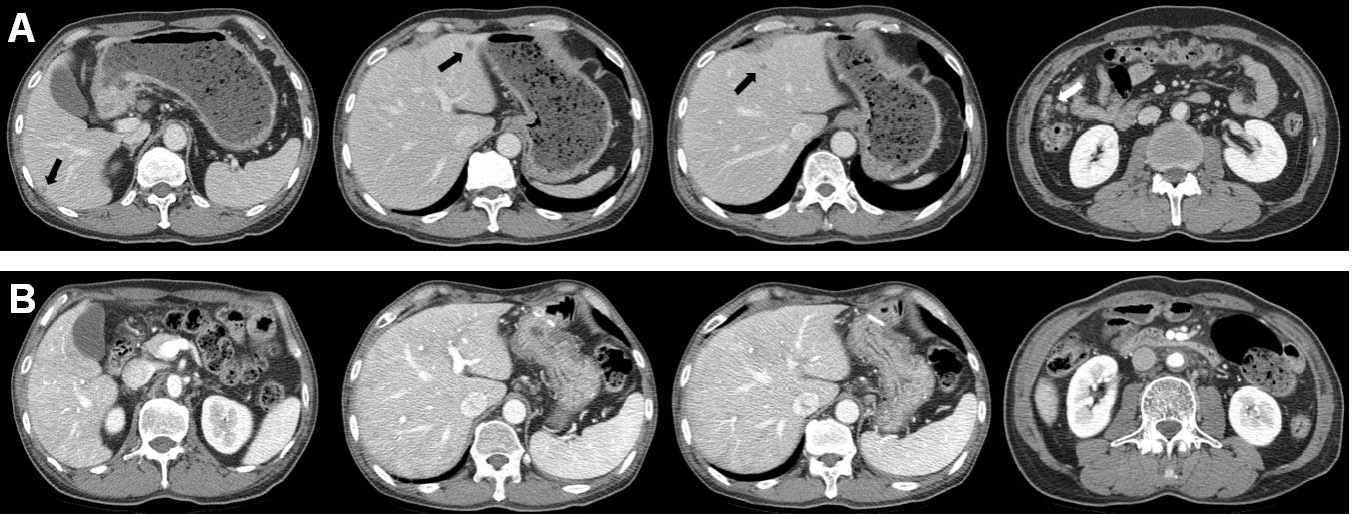
**Computed tomography (CT) scan (A) before and (B) after chemotherapy**. (A) A CT scan showed thickening of the gastric wall, adjacent fat infiltration, and a large amount of food in the stomach, suggesting a passage disturbance. Lymph nodes along the common hepatic and left gastric arteries were markedly enlarged, and multiple enhancing omental masses (white arrow) and nodular peritoneal thickening, suggesting peritoneal carcinomatosis, and multiple hepatic metastases (black arrows) are shown. (B) Complete disappearance of thickening of the gastric wall, the metastatic foci in the liver, omental infiltration of metastatic nodules, and enlarged lymph nodes were observed.

An abdominal computed tomography (CT) scan revealed thickening of the gastric wall, adjacent fat infiltration, and a large amount of food in the stomach, suggesting passage disturbance. The CT scan also showed enlarged lymph nodes along the common hepatic and left gastric arteries, multiple enhancing omental masses, nodular peritoneal thickening suggestive of peritoneal carcinomatosis, and multiple hepatic metastases (Figure 2A).

The serum carcinoembryonic antigen (CEA) level was 343 ng/ml and the carbohydrate antigen (CA) 19-9 level was within normal limits. The patient was diagnosed with unresectable gastric cancer with GOO and several incurable factors. The clinical stage was stage IV (cT4N2M1). The Eastern Cooperative Oncology Group (ECOG) performance status was grade 1 [6].

The patient underwent a laparoscopic gastrojejunostomy for palliation of the GOO. The peritoneal drainage catheter was placed during the laparoscopic procedure. During laparoscopic inspection, there were several metastatic nodules on the omentum. One of the nodules was biopsied and confirmed to be metastatic adenocarcinoma. On the 3^rd ^and 12^th ^days after surgery, for intraperitoneal chemotherapy, 40 mg of docetaxel and 150 mg of carboplatin were introduced over 2 hours in 1000ml of saline. Hyperthermia was not used in the intraperitoneal chemotherapy. Simultaneously, he received 85 mg/m^2 ^of oxaliplatin on the 1^st ^day as a 2-hour infusion followed by 600 mg/m^2 ^of 5-fluorouracil (FU) as a 22-hour infusion for 2 days (FOLFOX regimen) from the 8^th ^post-operative day repeated every 2 weeks.

After six courses, a CT scan revealed marked reduction in the size of the hepatic metastases, and the tumor markers returned to normal levels. After the completion of nine courses of chemotherapy, the gastric wall thickening, metastatic foci in the liver, omental infiltration of the metastatic nodules, and enlarged lymph nodes noted on CT scan resolved (Figure 2B). Positron emission tomography (PET) scan showed no metabolic evidence of malignancy. A gastroscopy showed an ulcerated scar in the antrum and the GOO and passage disturbance had also resolved (Figure 1B).

No malignant cells were detected on the endoscopic biopsy specimen, and a CR was determined according to the response evaluation criteria in solid tumors (RECIST) guideline[7].

He underwent a radical subtotal gastrectomy with a D3 lymph node dissection 4 months after the initial palliative surgery. The pathologic results showed no residual primary tumor and no lymph node metastasis of 43 dissected lymph nodes. There was no peritoneal metastatic focus in the surgical specimen including omentum and visceral peritoneum. Postoperatively, he received oral chemotherapy with daily 600 mg of doxifluridine for 12 months. He maintained a CR for 18 months after the last operation.

## Discussion

The prognosis of patients with highly advanced gastric cancer with distant metastasis, such as peritoneal dissemination or hematogenous metastasis, is usually very poor. When peritoneal dissemination is present, curative surgery cannot be achieved. However, aggressive treatment, including cytoreductive surgery with peritonectomy and intraperitoneal chemotherapy, have been reported for such patients [8]. Recently, Yonemura et al.[5] developed a new multimodal treatment referred to as bidirectional chemotherapy (neoadjuvant intraperitoneal-systemic chemotherapy protocol) for the treatment of peritoneal carcinomatosis [5]. They reported a 50% complete cytoreduction rate by cytoreductive surgery after neoadjuvant intraperitoneal and systemic chemotherapy. The median survival time of all 51 patients in that study was 14.4 months.

For bidirectional chemotherapy, various chemotherapeutic agents have been used for intraperitoneal and systemic chemotherapy [1,5]. For intraperitoneal chemotherapy in the present report, 40 mg of docetaxel and 150 mg of carboplatin were introduced, as described by Yonemura et al.[1].

For metastatic advanced gastric cancer, S-1 plus cisplatin was introduced as a standard treatment in Japan based on the randomized controlled trial [9]. Oxaliplatin has powerful anti-neoplasm activity, an intriguing alternative to cisplatin with at least comparable activity, a synergistic effect with 5-FU, and a satisfactory safety profile. The combination of oxaliplatin with 5-FU and leucovorin (FOLFOX regimen) was selected for systemic chemotherapy because it has a favorable activity as first-line therapy with locally advanced and metastatic gastric cancer or second-line treatment in advanced or metastatic gastric cancer patients, and may be considered a viable treatment alternative. This regimen was suggested to be active with a 40%-55% objective response rate in patients with gastric cancer [10-12]. However, this regimen has rarely been reported as a neoadjuvant chemotherapy agent in the treatment of advanced gastric cancer [13].

Neoadjuvant chemotherapeutic agents in the previous studies included S-1 [4,5], S-1 plus cisplatin [3], methotrexate plus 5-fluorouracil [1], paclitaxel plus doxifluridine [2], EEP (etoposide, epirubicin, and cisplatin) [14], and cisplatin plus 5-fluorouracil with leucovorin [15]. The FOLFOX regimen has usually been used for palliative chemotherapy for patients with metastatic gastric cancer. We used the FOLFOX regimen as neoadjuvant chemotherapy, which is widely used for metastatic advanced gastric cancer after curative or palliative resection or for unresectable gastric cancer in our institute.

In the present case, the patient had several incurable factors, including peritoneal carcinomatosis, hepatic metastasis, and locally advanced tumor that induced GOO. Therefore, the first aim of treatment was palliation of the GOO symptoms. We performed laparoscopic bypass surgery first, and at that time, an intraperitoneal port was implanted for the following intraperitoneal chemotherapy.

GOO is a challenging problem in patients with advanced gastric cancer in the distal part of the stomach. The presence of GOO is an independent prognostic factor, even after radical surgery [16]. Additionally, for systemic chemotherapy, GOO is a problem that should be treated first because adequate oral intake is essential for systemic chemotherapy. For GOO, various treatment options, such as endoscopic stenting, palliative bypass surgery (open or laparoscopic), or palliative resection, can be chosen. Resection is theoretically the most effective treatment option for GOO by achieving intestinal continuity and a reductive therapeutic effect. In the present case, however, other incurable factors existed, such as hepatic metastasis and peritoneal carcinomatosis. Furthermore, palliative bypass surgery (gastrojejunostomy) is thought to provide better long-term results compared to endoscopic stent placement, and is therefore the treatment of choice in patients with a life expectancy of > 2 months [17]. Thus, we performed laparoscopic palliative gastric bypass surgery rather than palliative resection or stent placement.

If curative resection is not possible or not effective, and response to chemotherapy is expected, laparoscopic palliative procedure is an effective and safe procedure option for the patient with GOO because this palliative procedure is minimally invasive and enable the patient to receive early post-operative chemotherapy. In addition, the port for the intraperitoneal chemotherapy can be placed during this procedure. Furthermore, laparoscopic bypass surgery can prevent post-operative adhesions and enable easy, definitive surgery for the patient who has a response to the chemotherapy.

For the second operation after CR was suspected by image study and endoscopic biopsy, we performed radical subtotal gastrectomy with extended lymph node dissection. The aim of the surgical resection was to confirm the CR by pathologic examination and to provide the therapeutic effect of resection if the residual cancer cells were present. However, hepatic resection was not performed because the initial hepatic metastasis was multiple and bilobar, and the location of hepatic metastasis could not be identified at the time of the second operation.

According to previous reports regarding neoadjuvant chemotherapy for advanced gastric cancer, aggressive treatment for peritoneal dissemination has been limited to patients with peritoneal carcinomatosis alone, rather than hematogenous metastasis, such as hepatic metastasis or distant lymph node metastasis [1,5,8,13]. D'Ugo et al. [14] reported neoadjuvant chemotherapy in patients with resectable gastric cancer. However, in the present case, a CR was observed in spite of the fact that he had three individual incurable factors; peritoneal dissemination, hepatic metastasis, and GOO caused by locally advanced primary tumor. The reason for the good response of our case may be related to the following: 1) the size of the multiple hepatic metastases was small; 2) peritoneal dissemination was limited to the omentum and not disseminated to the distant peritoneum; and 3) the tumor size was relatively small in spite of GOO, and there was no adjacent organ invasion (pancreas or colon). Thus, to evaluate the effectiveness of chemotherapy following bypass surgery for unresectable gastric cancer with GOO, a large randomized controlled trial is needed.

## Conclusion

Combination chemotherapy with systemic and intraperitoneal chemotherapy following laparoscopic bypass surgery showed marked efficacy in the treatment for unresectable advanced gastric cancer with GOO.

## Consent

Written informed consent was obtained from the patient for publication of this case report and accompanying images. A copy of the written consent is available for review by the Editor-in-Chief of this journal.

## Competing interests

The authors declare that they have no competing interests.

## Authors' contributions

JP and KC equally contributed to this study and were responsible for treatment of the case and writing the manuscript. All authors read and approved the final manuscript.
